# Spontaneous multi-focal coronary artery spasm: a case report

**DOI:** 10.15171/jcvtr.2016.28

**Published:** 2016-09-25

**Authors:** Mohammad Ali Ostovan, Mahdi Sajedi Khanian, Sahand Hamidi, Mostafa Fattahi, Pooyan Dehghani

**Affiliations:** ^1^Cardiology Department, School of Medicine, Shiraz University of Medical Sciences, Shiraz, Iran; ^2^Cardiovascular Research Center, Shiraz University of Medical Sciences, Shiraz, Iran

**Keywords:** Coronary Spasm, Myocardial Infarction, Intracoronary Nitroglycerin

## Abstract

Spontaneous coronary artery vasospasm is one of the important causes of acute chest pain syndromes. The diagnosis of diffuse multifocal spasm can be quite challenging and it could be easily mistaken for diffuse coronary artery disease. The use of intracoronary nitroglycerin can relieve spasm and reveal the real extent of coronary artery disease. Herein we present a case presenting with acute myocardial infarction due to severe coronary artery spasm that had even received fibrinolytic therapy. Multiple narrowing was shown during coronary angiography and the patient was scheduled for percutaneous coronary intervention (PCI). But after intracoronary (IC) injection of nitroglycerin, all of lesions disappeared completely and the diagnosis of coronary spasm was confirmed.

## Introduction

Spontaneous coronary artery vasospasm is one of the important causes of acute chest pain syndromes.^1^ It can lead to myocardial infarction, arrhythmias, sudden cardiac death and even unnecessary procedures including percutaneous coronary intervention (PCI) and coronary artery bypass graft surgery.^2^ Herein we discuss an interesting case of anterolateral ST elevation myocardial infarction which was finally diagnosed as multifocal spontaneous coronary artery spasm after several inappropriate decision makings.

## Case presentation

**Figure 1:**
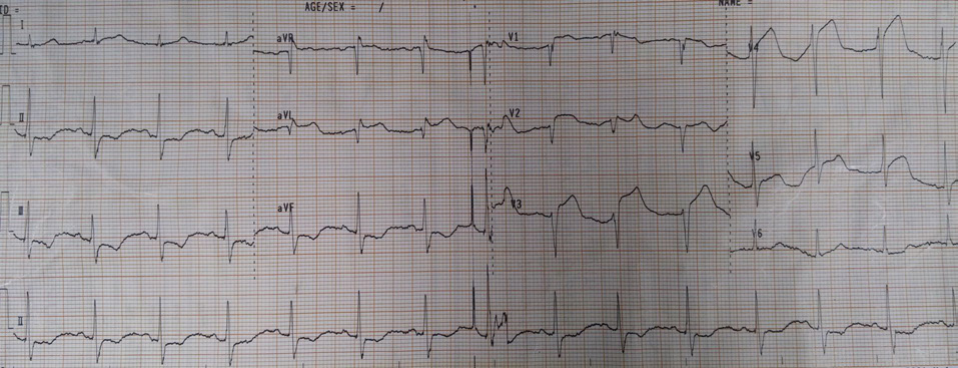
Electrocardiogram of the patient.

A 45-year-old man presenting with typical retrosternal chest discomfort received fibrinolytic therapy with the impression of acute anterolateral ST elevation myocardial infarction (Figure 1). He had history of asthma and cigarette smoking but there was no history of illicit drugs or alcohol abuse. He was transferred to our center for an early invasive strategy.

On admission, vital signs were stable and physical examination was unremarkable except for the presence of S4 and a mild diffuse end-expiratory wheezing.

Laboratory data showed mild leukocytosis and a high troponin I level. In echocardiographic evaluation there was mild left ventricular systolic dysfunction with ejection fraction of 45% associated with anterior and anterolateral wall hypokinesia and a mild mitral regurgitation. The patient was scheduled for cardiac catheterization which was 5 hours after receiving thrombolytic therapy.

**Figure 2:**
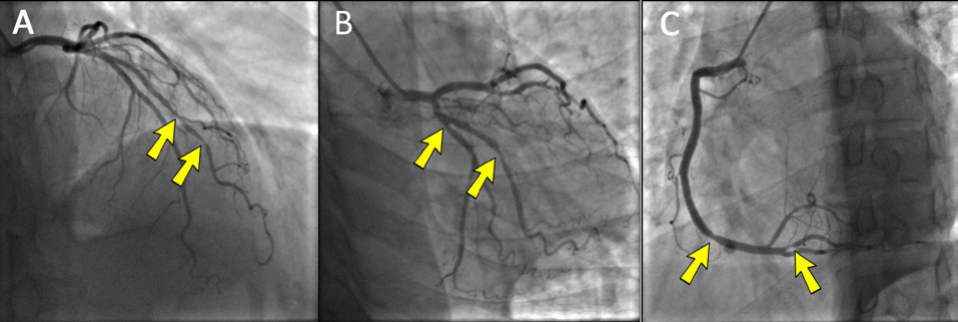
Coronary angiogram of the patient, Panel A: Left coronary injection, significant narrowing of large diagonal branch (arrows), Panel B: Left coronary injection, narrowing of left circumflex artery and severe narrowing of first obtuse branch artery (arrows). Panel C: Right coronary injection, narrowing of right coronary artery in distal part and posterior descending artery (arrows)

Coronary angiography was performed via right radial access and the findings were: normal left main trunk and left anterior descending artery (LAD), first diagonal (D1) was large with significant midpart narrowing at 2 sites (Figure 2, Panel A, Arrows), moderate midpart narrowing of left circumflex artery (LCx), severe narrowing of first obtuse marginal branch (OM1) (Figure 2 Panel B, Arrows), Mild to moderate narrowing of distal right coronary artery, significant narrowing posterior descending artery (PDA) (Figure 2, Panel C, Arrows).

**Figure 3:**
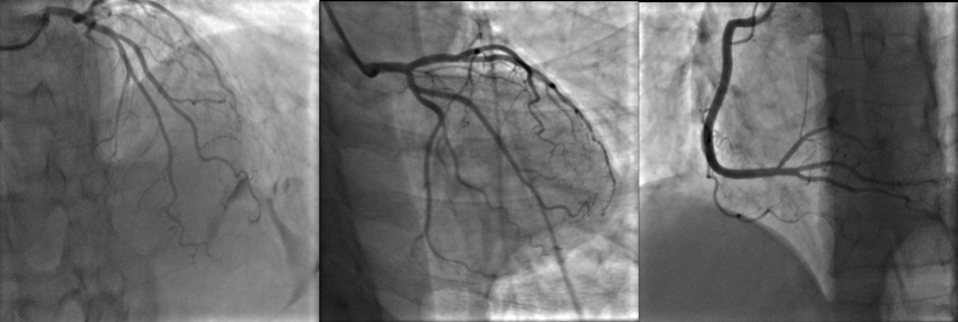
Coronary angiogram after administration of intra coronary nitroglycerin.

After discussing the situation, we planned for multivessel PCI on D1, OM and PDA. D1 lesion was targeted first but after intracoronary (IC) injection of nitroglycerin, the lesion disappeared completely. The whole study was repeated after IC nitroglycerin administration and the result was patent coronary arteries without any narrowing (Figure 3). So, diagnosis of multifocal spontaneous coronary spasm was confirmed, procedure was terminated and medical therapy with long acting nitrates and calcium channel blockers was initiated. The patient was discharged after 2 days with no complications.

## Discussion

Spontaneous coronary artery vasospasm is described as a transient narrowing or occlusion of an artery. It has the incidence of 0.3-3% during coronary angiography and is an important cause of morbidity.^1,3^ Endothelial dysfunction seems to be the most important contributing factor.^4^ Although patients with coronary spasm generally have a good prognosis even during acute coronary syndromes but the presence of multivessel coronary spasm could be a predictor of adverse prognosis.^5^

Multivessel coronary spasm is very uncommon and normal coronary vasculature is present in one third.^6^ It could be mistaken for diffuse obstructive atherosclerotic disease^2,6,7^ and result in unnecessary revascularization therapies such as coronary artery bypass grafting (CABG) or PCI.^2,7^

The most common manifestations of coronary spasm are related to episodic periods of myocardial ischemia which can lead to myocardial infarction, malignant arrhythmias and even sudden cardiac death in severe forms. Usually the only atherosclerotic risk factor is cigarette smoking.^1,4,6^

One of the important issues is to distinguish between spontaneous coronary spasm and catheter induced spasm. Spontaneous coronary spasm can occur in any coronary artery and far from the catheter tip in different lengths. It has irregular border and is eccentric. Finally it is associated with angina, hypotension, arrhythmia, and ST segment elevation in electrocardiogram. In contrast catheter induced spasm is often asymptomatic and occurs at the catheter tip and usually in right coronary artery. Its shape is almost invariably concentric with smooth borders and less than 2 mm in length.^8^

Diffuse multifocal spasm diagnosis can be quite challenging and could be easily mistaken for diffuse coronary artery disease.^2,6,7^ There are some case reports in the literature that invasive procedures are performed for these patients. Ahooja and Thatai presented a case of diffuse multi vessel vasospasm resulting in CABG because it was misdiagnosed as atherosclerotic obstructive coronary artery disease. The only risk factor they could find was a positive urine screening test for opiates and benzodiazepines, but not for cocaine or its metabolites.^2^ Another report by Guardado et al was a case of two vessel disease that presented with cardiogenic shock immediately after adhoc PCI due to diffuse coronary spasm. The patient was a 50-year-old man, smoker and hypertensive, with chronic renal failure on routine dialysis, without history of cocaine or other illicit drug abuse.^7^ It seems that smoking plays a role in multifocal coronary spasm in these cases as well as our case.

Our patient presented with acute myocardial infarction due to severe coronary artery spasm and even received fibrinolytic therapy. Multiple narrowing was shown during coronary angiography and the patient was scheduled for PCI. If IC nitroglycerin was not administered before angioplasty the serial of unnecessary managements for this patient could be completed (e.g. thrombolytic therapy and PCI).

It is suggested to administer intracoronary nitrates in every patient with the suspicion of coronary spasm before terminating the diagnostic assessment of coronary arteries. Maybe routine injection of IC nitroglycerin before angioplasty procedure would be helpful in diagnosing some cases of coronary artery spasms. It could help to evaluate the extent of coronary disease better and aid the physician for optimal management and prevent unnecessary high risk interventions. This case highlights the value of intracoronary nitroglycerin injection before angioplasty.

## Conclusion

Occasionally, vasospasm can occur extensively either in the presence or absence of obstructive lesions and pose a serious therapeutic dilemma. The use of intracoronary nitroglycerin can relieve spasm and reveal the real extent of coronary artery disease. Therefore appropriate use of nitroglycerin and heightened clinical suspicion could impede unnecessary interventions and help in the optimal therapy for these cases.

## Ethical approval

This study was approved by the ethics committee, Shiraz University of Medical Sciences, Shiraz, Iran. Informed consent form was signed by the patient.

## Competing interests

All authors declare no competing financial interests exist.
